# The effect of unmet needs on the health-related Quality of life of family caregivers of cancer patients in South Korea

**DOI:** 10.1371/journal.pone.0321900

**Published:** 2025-05-02

**Authors:** Juyeun Kim, Sangmi Lee

**Affiliations:** College of Nursing, Dongyang University, Yeongju-si, Gyeongsangbuk-do, Republic of Korea; Prince Sattam bin Abdulaziz University, SAUDI ARABIA

## Abstract

This study was conducted to explore the unmet needs and health-related quality of life (QOL) of family caregivers who support cancer patients, and to determine the impact of these unmet needs on their health-related QOL. A descriptive study was conducted from July 1 to July 30, 2023, in which a survey was administered to 129 family caregivers of cancer patients undergoing outpatient treatment at a general hospital of South Korea. The effect of unmet needs on health-related QOL was analyzed using stepwise multiple regression analysis, while controlling for covariates such as the general characteristics of the participants. Stepwise multiple regression analysis revealed that unmet needs significantly impacted health-related QOL. The final regression model explained a substantial portion of the variance in health-related QOL, with an R-squared value of.466 (46.6%). Unmet needs of health and psychological problems (β = -.37) as well as religious/spiritual support (β = -.20) had a significant independent effect on the health-related QOL of family caregivers of cancer patients. Additionally, the health-related QOL of caregivers was significantly better when they did not live with the patient (β = .29) and when they had higher income levels (β = .18) for incomes between 300–500 million won, and β = .29 for incomes of 500 million won or more, compared to those earning less than 100 million won). The health-related QOL of family caregivers for cancer patients can be adversely affected by unmet needs, including health and psychological problems as well as religious and spiritual support. It is therefore necessary to develop and implement support programs or systems that promote physical, psychological, and spiritual health for these caregivers.

## Introduction

The cancer incidence rate in South Korea in 2021 was 540.6 per 100,000 people. If individuals live to the average life expectancy of 83.6 years, the likelihood of developing cancer is estimated to be 38.1% [1]. Furthermore, thanks to advancements in early diagnosis and treatment technologies, the survival rates for cancer patients have been steadily improving. The 5-year relative survival rate for cancer patients between 2017 and 2021 was 72.1%. The rapid increase in the number of cancer patients is attributed to both the rise in cancer incidence and the improved survival rates [1]. This trend underscores the growing importance of caregivers in supporting cancer patients through treatment and continued care.

Especially, the symptoms and treatment process of cancer vary according to its stage, and the chronic and uncertain prognosis of the disease presents significant challenges for both patients and caregivers [2,3]. Family members of cancer patients are tasked with providing physical, social, and financial support throughout the treatment process [2]. They must adapt to various roles from the time of diagnosis through the progression of the disease to palliative care, depending on the stage of the illness [4]. Moreover, with hospital stays for cancer treatment becoming shorter, caregivers are increasingly responsible for tasks such as medication administration and symptom management, which were traditionally handled by medical staff with specialized knowledge and skills [3,5]. A previous study revealed that caregivers of cancer patients in the United States devote an average of 32.9 hours per week to caregiving, with 72% performing complex medical or nursing tasks [6].

With the increasing burden of caregiving for cancer patients, attention to the unmet needs of caregivers is growing, and this topic is being discussed as an important area of research. While caregiver burden refers to the extent to which caregivers perceive that their overall well-being is compromised due to physical fatigue, psychological stress, limitations in social activities, and financial difficulties resulting from caring for a cancer patient [7], unmet needs refer to the state in which caregivers lack the necessary support, such as information, financial assistance, and psychological support, required to effectively provide care or maintain their own lives [8]. Thus, unmet needs are distinct from caregiver burden. For example, if a caregiver is struggling to manage a patient’s symptoms but does not receive adequate education or support, this can be considered an unmet need. Numerous previous studies have reported that if these unmet needs persist, they can increase caregiver burden and burnout, leading to a decline in their quality of life (QOL) [9,10].

Previous research has shown that caregivers of breast cancer patients with lower QOL experience higher levels of daily and despair stress, correlating with greater unmet needs [3]. Conversely, caregivers of patients with end-stage ovarian cancer reported a higher QOL when their needs were met [8]. Additionally, unmet needs among caregivers can lead to a lack of confidence and caregiving capacity, financial constraints, misuse of information, and communication difficulties with service providers, all of which hinder their ability to deliver quality care to patients [4]. A study by Janda et al. [11] found a correlation between anxiety and depression symptoms in both patients and caregivers, suggesting that the health-related QOL and psychological well-being of caregivers can also influence patients. Therefore, exploring the unmet needs of family caregivers of cancer patients is a critical step not only in improving their QOL but also in delivering high-quality medical services to patients.

In Korean society, a strong traditional familial culture persists, where family members are expected to care for their ill relatives [12,13]. However, support policies for end-of-life patients in Korea primarily focus on inpatient services, while home care services are mainly provided to dementia patients. **This approach fails to adequately address the needs of family caregivers of cancer patients who prefer home-based care** [14]. In contrast, while caregiving for cancer patients is also recognized as a significant issue in the United States, home hospice services are widely available. Various support systems, such as inpatient respite care and hospice aids & homemaker services, are effectively utilized, contributing to the reduction of caregiver burden [14]. Consequently, family caregivers in Korea often sacrifice their own health and QOL to provide care without sufficient access to formal support systems [12]. This situation can lead to deterioration in the physical and mental health of caregivers and ultimately negatively impact the patient’s health as well [11]. Therefore, it is crucial to identify the unmet needs of family caregivers in Korea and develop systematic support policies that consider cultural characteristics.

However, recent research has primarily focused solely on cancer patients to identify their unmet needs [15,16]. Additionally, there have been studies targeting the unmet needs of families, predominantly those with hospitalized patients [13,17,18]. However, as the treatment paradigm for cancer patients shifts towards outpatient care, these earlier studies may not adequately capture the diverse needs of family caregivers who provide support at home. Therefore, this study is designed to elucidate the complex interplay between unmet needs and health-related QOL in Korean family caregivers of cancer patients. Specifically, it aims to: (1) identify the unmet needs and health-related QOL experienced by family caregivers of cancer patients in Korea; (2) analyze the differences in unmet needs and QOL according to the characteristics of the subjects; (3) determine the specific impact of unmet needs on health-related QOL. The results obtained through this research will provide crucial evidence for developing effective caregiving support policies and programs tailored to the Korean context.

## Methods

### Study design

This descriptive study aimed to examine the unmet needs and health-related QOL of family caregivers of cancer patients, and to investigate the impact of unmet needs on their health-related QOL. A descriptive study approach is ideal for systematically characterizing a specific population and quantifying the magnitude of particular phenomena. In this case, it allowed us to quantitatively assess the levels of unmet needs and QOL among Korean family caregivers, and to delineate the relationship between these variables.

### Study participants

The target population of this study comprised family care providers for cancer patients under outpatient-centered treatment, and this study used convenience sampling to recruit family care providers visiting the hematologic oncology department of hospital A located in K province with cancer patients. Participants were family caregivers over the age of 20 whose relatives were receiving outpatient chemotherapy or cancer-related treatment in hematologic oncology. Additionally, to ensure homogeneity of the study population, caregivers of patients diagnosed with cancer more than five years prior were excluded. This decision was based on the 5-year relative survival rate, a standard metric used by major health organizations to assess cancer treatment outcomes. The 5-year relative survival rate is defined as the probability of a cancer patient surviving for at least five years, adjusted for the possibility of death from other causes [1]. After this period, the risk of cancer recurrence typically declines, and the associated caregiving demands often shift, potentially introducing confounding factors into the analysis.

The number of samples in this study was calculated using G*Power 3.1.9, 127 people were calculated when the effect size for multiple regression was.15, significance level.05, power.80, and the number of independent variables was 12. In consideration of the dropout rate such as unfaithful responses or refusal to participate, a total of 150 people were collected, and a total of 129 people were included in the final subject, excluding 21 people who responded unfaithfully to the survey.

The general characteristics of the study participants are presented in Table 2. The mean age of the participants was 49.76 ± 16.96 years, with the largest age group being those in their 60s or older (n = 70, 54.3%), comprising 70 individuals (54.3%). There were more women (n = 85; 65.9%) than men. Most of the participants were married (n = 95; 73.6%). Sixty-four (49.6%) participants reported being religious. Educational attainment varied, with 53 participants (41.1%) having completed college or higher, 47 (36.4%) having finished high school, and 29 (22.5%) having an education level of middle school or lower. Eighty-one participants (62.8%) were employed. The largest category of monthly income was 5 million won or more (n = 51; 39.5%), while the smallest group comprised those earning less than 1 million won (n = 17; 13.2%), earning less than 1 million won. Eighty participants (62.0%), lived with the patients, with spouses being the most common caregivers (n = 54; 41.9%), followed by parents (n = 31; 24.0%), children (n = 30; 23.2%), and other relatives (n = 14; 10.9%). Lung cancer was the most common diagnosis, affecting 34 patients (26.4%), followed by a category labeled “others” with 31 patients (24.0%), and colorectal cancer with 16 patients (12.4%). The average duration of illness among the patients was 27.30 ± 18.68 months, with the most common duration being between 13 and 24 months (n = 43; 33.3%). The distribution of cancer stages among patients was relatively even: 35 patients (27.1%) were at stage 1, 38 (29.5%) at stage 2, and 33 (25.6%) at stage 3.

### Measurements

#### Unmet needs.

In this study, the unmet needs of caregivers were assessed using the Comprehensive Needs Assessment Tool for Cancer-caregivers (CANT-C), developed by Shin et al. [19]. The CANT-C comprises 41 items across seven domains: health and psychological problems (6 items), family/social support (5 items), healthcare staff (8 items), Information (8 items), religious/spiritual support (2 items), hospital facilities and services (4 items), and practical support (8 items). Each item is rated on a 4-point Likert scale, ranging from 0 (“no need”) to 3 (“much need”), with higher scores indicating greater unmet needs. At the time of its development, the CANT-C demonstrated a Cronbach’s α of.96 [19]. In the current study, the overall Cronbach’s α was.97, with the α values for the subdomains ranging from.60 to.96.

#### Health-related QOL.

In this study, health-related QOL was measured using the Korean version of the WHOQOL-BREF, which was standardized by Min et al. [20]. The WHOQOL-BREF is a World Health Organization Quality-of-Life Scale that includes a total of 26 items: two items address overall QOL, and the remaining 24 items are divided into four domains—seven items in the physical domain, six in the psychological domain, three in the social relationships domain, and eight in the environmental domain. Responses to each item were recorded on a 5-point Likert scale, ranging from 1 (“not at all”) to 5 (“very much so”). To facilitate comparisons between domains, scores for each area must be transformed into a scale ranging from 4 to 20 points, with higher scores indicating a better QOL. Cronbach’s α for the instrument was reported as.898 in the standardization study by Min et al. [20], and in the current study, it was.94. The Cronbach’s α values for the subdomains were as follows:.88 for overall QOL,.66 for physical health,.74 for psychological health,.81 for social relationships, and.90 for the environment.

### Data collection

The data for this study were collected using a questionnaire from July 1 to July 30, 2023, following approval from the Institutional Review Board (approval number: 1041495–202108-HR-01–01). To recruit participants, an official letter containing the research plan was sent to the head of a general hospital in South Korea. Approval was granted following a detailed explanation of the study’s purpose, methodology, participant selection criteria, and data collection procedures. The survey was administered by two research assistants who had undergone training provided by the lead researcher. The structured questionnaire survey was administered in a face-to-face interview format to family caregivers of patients waiting for outpatient treatment. Before the survey began, the purpose and methods of the study, as well as assurances of participant anonymity, were thoroughly explained, and informed consent has been voluntarily obtained from all participants. They were also clearly informed that there would be no disadvantages if they chose to withdraw from the study. Participant confidentiality and data security were rigorously maintained throughout the study. All questionnaires were anonymized to remove any identifying information before being coded and entered into a password-protected database. The researcher conducted all statistical analyses using only the coded data. Original paper questionnaires were stored separately in a locked cabinet within a secure office, and electronic data was stored on an encrypted external hard drive with restricted access limited to authorized research personnel. All research data will be archived for three years following publication of the study findings, after which it will be permanently deleted following a secure data disposal protocol. The research data will not be used for any purposes beyond the scope of this study, and the privacy of research participants was of utmost importance.

### Data analysis

The collected data were analyzed using SPSS Statistics 28.0 (IBM Corp., Armonk, NY, USA). Descriptive statistics, including frequency, percentage, mean, and standard deviation, were employed to characterize the general attributes, unmet needs, and health-related QOL of the participants. To assess the differences in unmet needs and health-related QOL according to the participants’ general characteristics, the independent t-test and one-way ANOVA were utilized, with the Scheffé method applied for post-hoc testing. Pearson’s correlation coefficient was used to determine the relationship between unmet needs and health-related QOL. Additionally, to examine the impact of unmet needs on health-related QOL, and to identify the most significant predictors, stepwise multiple regression analysis was employed. This method allows for the stepwise inclusion of independent variables, facilitating a detailed evaluation of each variable’s unique contribution to the explained variance in health-related QOL. Lastly, the interaction effects influencing the health-related QOL of the study participants were examined using Model 1 of the PROCESS macro by Hayes [21].

## Results

### Unmet needs and health-related QOL of the participants

**Fig 1:**
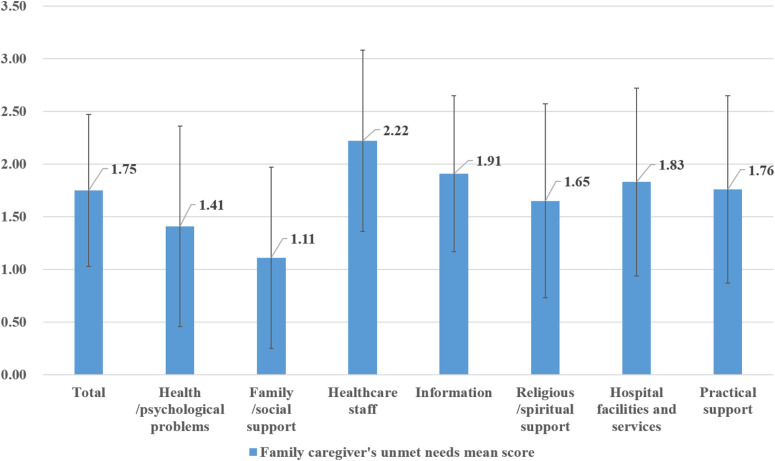
Unmet needs of the participants.

The unmet needs and health-related QOL levels of the participants are presented in Table 1. The average score for unmet needs was 1.75 ± 0.72, with “healthcare staff” scoring the highest at 2.22 ± 0.86. This was followed by “information” at 1.91 ± 0.74 and “hospital facilities and services” at 1.83 ± 0.89. In contrast, “family/social support” scored the lowest at 1.11 ± 0.86 (Fig 1). The average score for health-related QOL was 3.08 ± 0.83.

**Table 1:** Unmet needs and health-related quality of life of the participants.

Variables	M ± SD	Minimum	Maximum	Kurtosis	Skewness
**Unmet needs**	1.75 ± 0.72	0.13	2.93	-0.42	-0.92
**Health/psychological problems**	1.41 ± 0.95	0.00	3.00	0.08	-1.35
**Family/social support**	1.11 ± 0.86	0.00	3.00	0.13	-1.26
**Healthcare staff**	2.22 ± 0.86	0.13	3.00	-0.94	-0.22
**Information**	1.91 ± 0.74	0.25	3.00	-0.51	-0.85
**Religious/spiritual support**	1.65 ± 0.92	0.00	3.00	-0.12	-0.82
**Hospital facilities and services**	1.83 ± 0.89	0.00	3.00	-0.41	-0.91
**Practical support**	1.76 ± 0.89	0.00	3.00	-0.29	-1.04
**Health-related quality of life**	3.08 ± 0.83	1.15	4.96	0.01	-0.49

### Differences in unmet needs and health-related QOL according to the general characteristics of participants

Table 2 shows the differences in unmet needs and health-related QOL based on the general characteristics of the participants. Unmet needs were statistically significantly greater among participants with an educational level of under middle school graduation (F = 3.95, p = .022) than those with education beyond high school. Additionally, unmet needs were higher when the cancer stage was 3 than when it was 1 (F = 6.41, p < .001).

**Table 2:** Differences in unmet needs and health-related quality of life according to the general characteristics of the participants (N = 129).

Variables	Categories	n (%) or M ± SD	Unmet needs		Health-related QOL	
			M ± SD	t or F (p)	M ± SD	t or F (p)
***Family caregivers of patients***						
**Ages**	20s and 30s	25 (19.4)	1.47 ± 0.76	2.68 (.072)	3.49 ± 0.83	7.95 (.001) c < a
	40s and 50s	34 (26.4)	1.88 ± 0.67		3.29 ± 0.76	
	60s and older	70 (54.3)	1.78 ± 0.72		2.83 ± 0.78	
	Total	49.76 ± 16.96				
**Gender**	Male	44 (34.1)	1.63 ± 0.72	-1.30 (.197)	3.08 ± 0.75	-0.03 (.979)
	Female	85 (65.9)	1.81 ± 0.72		3.08 ± 0.87	
**Marital status** ^*^	Unmarried/divorced /separated	34 (26.4)	1.55 ± 0.72	-1.89 (.061)	3.39 ± 0.83	2.59 (.011)
	Married	95 (73.6)	1.82 ± 0.71		2.97 ± 0.80	
**Religious**	Yes	64 (49.6)	1.85 ± 0.69	1.59 (.113)	2.97 ± 0.87	-1.52 (.132)
	No	65 (50.4)	1.65 ± 0.74		3.19 ± 0.78	
**Academic background** ^*^	Middle school^a^	29 (25.5)	2.07 ± 0.58	3.95 (.022) b,c < a	2.30 ± 0.59	22.20 (<.001) a < b,c
	High school^b^	47 (36.4)	1.65 ± 0.72		3.25 ± 0.81	
	≥College^c^	53 (41.1)	1.66 ± 0.75		3.35 ± 0.69	
**Employed**	Yes	81 (62.8)	1.71 ± 0.73	-0.71 (.479)	3.06 ± 0.77	0.19 (.851)
	No	48 (37.2)	1.81 ± 0.71		3.06 ± 0.93	
**Monthly income level** **(million** **won)** ^*^	<100^a^	17 (13.2)	1.97 ± 0.73	.845 (.472)	2.45 ± 0.74	7.30 (<.001) a < c,d
	≥100, < 300^b^	29 (22.5)	1.62 ± 0.74		2.83 ± 0.90	
	≥300, < 500^c^	32 (24.8)	1.75 ± 0.79		3.19 ± 0.61	
	≥500^d^	51 (39.5)	1.75 ± 0.66		3.36 ± 0.80	
**Living with patient**	Yes	80 (62.0)	1.74 ± 0.76	-0.18 (.858)	2.83 ± 0.81	-4.68 (<.001)
	No	49 (38.0)	1.76 ± 0.66		3.48 ± 0.70	
**Relationship with patient** ^*^	Spouse^a^	44 (34.1)	1.75 ± 0.74	0.16 (.921)	2.65 ± 0.81	7.44 (<.001) a < c,d
	Child^b^	30 (23.3)	1.68 ± 0.14		3.20 ± 0.78	
	Parent^c^	41 (31.8)	1.77 ± 0.70		3.31 ± 0.70	
	Relative^d^	14 (10.9)	1.83 ± 0.68		3.51 ± 0.83	
***Patients***						
**Types of cancer**	Stomach cancer	12 (9.3)	2.19 ± 0.51	2.17 (.051)	2.78 ± 0.93	1.21 (.307)
	Lung cancer	34 (26.4)	1.89 ± 0.69		2.97 ± 0.85	
	Liver cancer	14 (10.9)	1.69 ± 0.75		3.14 ± 0.84	
	Colon cancer	16 (12.4)	1.67 ± 0.78		2.91 ± 0.80	
	Breast cancer	12 (9.3)	1.31 ± 0.67		3.47 ± 0.81	
	Kidney cancer	10 (7.8)	1.44 ± 0.72		2.98 ± 0.66	
	Others	31 (24.0)	1.76 ± 0.72		3.26 ± 0.82	
**Duration of cancer (months)**	≤12	31 (24.0)	1.62 ± 0.83	0.95 (.417)	3.15 ± 0.71	1.44 (.234)
	≥13, ≤ 24	43 (33.3)	1.69 ± 0.76		3.03 ± 0.85	
	≥25, ≤ 36	17 (13.2)	1.84 ± 0.65		3.41 ± 1.04	
	≥37, ≤ 60	38 (29.5)	1.88 ± 0.60		2.93 ± 0.77	
	Total	27.30 ± 18.68			3.15 ± 0.71	
**Stage of cancer** ^*^	Don’t know^a^	5 (3.9)	1.82 ± 0.39	6.4 1(<.001) b < d	3.48 ± 0.91	1.11 (.355)
	1^b^	35 (27.1)	1.33 ± 0.75		3.24 ± 0.65	
	2^c^	38 (29.5)	1.71 ± 0.72		3.09 ± 0.90	
	3^d^	33 (25.6)	2.11 ± 0.56		2.94 ± 0.82	
	4^e^	18 (14.0)	1.95 ± 0.61		2.88 ± 0.97	

*Scheffé method

Health-related QOL was highest among individuals in their 20s and 30s, showing a statistically significant difference compared to those aged 60 or older (F = 7.95, p = .001). Furthermore, QOL was higher among married individuals compared to their non-married counterparts (F = 2.59, p = .011), and among those with a higher education level as opposed to those with a middle school education or lower (F = 22.20, p < .001).

### Correlation between unmet needs and health-related QOL

Table 3 shows the correlations between participants’ unmet needs and health-related QOL. There was a significant negative correlation between unmet needs and health-related QOL (r = -.45, p < .001). In addition, the sub-areas of unmet needs—health and psychological problems (r = -.49, p < .001), family/social support (r = -.41, p < .001), healthcare staff (r = -.20, p = .026), information (r = -.35, p < .001), religious/spiritual support (r = -.38, p < .001), hospital facilities and services (r = -.37, p < .001), and practical support (r = -.45, p < .001)—all showed significant negative correlations with health-related QOL.

**Table 3:** Correlations between unmet needs and health-related quality of life.

Variables	Health-related quality of life, r (p)
**Unmet needs**	-.45 (<.001)
**Health/psychological problems**	-.49 (<.001)
**Family/social support**	-.41 (<.001)
**Healthcare staff**	-.20 (.026)
**Information**	-.35 (<.001)
**Religious/spiritual support**	-.38 (<.001)
**Hospital facilities and services**	-.37 (<.001)
**Practical support**	-.45 (<.001)

### Factors influencing health-related QOL

Table 4 presents the results of stepwise multiple regression analysis on the factors affecting the health-related QOL of family caregivers of cancer patients. Before conducting the multiple regression analysis, we assessed multicollinearity by checking the tolerance, variance inflation factor (VIF), and performing the Durbin-Watson test. The tolerance values ranged from 0.41 to 0.96, with no variables falling below 0.1, and the VIF values were between 1.04 and 2.43, which is well below the threshold of 10. Additionally, the Durbin-Watson statistic was 1.84, indicating no issues with autocorrelation of residuals. In the regression model, we included unmet needs, as the primary variable of this study, along with age, marital status, education, income level, cohabitation with patients, and relationship to the patient. These variables demonstrated statistically significant associations with health-related QOL in the univariate analysis. They were incorporated as covariates, transformed into dummy variables, and entered into the regression model.

**Table 4:** Factors affecting health-related quality of life.

Variables	B	SE	β	t (p)	R^2^	△R^2^
**Constant**	3.38	0.14				
**Health and psychological problems**	-0.32	0.07	-.37	-4.85 (<.001)	.238	.238
**Living with patients (no)** ^*^	0.50	0.12	.29	4.14 (<.001)	.377	.138
**Income level (**≥5 million won)^*^	0.49	0.13	.29	3.71 (<.001)	.410	.034
**Religious/spiritual support**	-0.19	0.07	-.20	-2.70 (<.001)	.442	.032
**Income level (**≥3 and < 5 million won)^*^	0.35	0.15	.18	2.34 (.021)	.466	.024

*Dummy variables of reference groups: income level (<1 million won), living with patients (yes)

The analysis revealed that unmet needs for health and psychological problems significantly impacted the QOL of family caregivers of cancer patients, accounting for 23.8% (R^2 ^= .238) of the variance. The inclusion of “living together (no)” as a variable increased the explanatory power by 13.8%, bringing it to 37.7% (R^2 ^= .377). Adding “income level (more than 5 million won) “ further raised the explanatory power by 3.4% to a total of 41.0%. The consideration of “religious/spiritual support” contributed an additional 3.2% increase, resulting in an explanatory power of 44.2% (R^2 ^= .442). Finally, incorporating “income level (3 to less than 5 million won) “ led to a 2.4% increase, culminating in an overall explanatory power of 46.6% (R^2 ^= .466) for the regression model. In this study, a higher income level, specifically 5 million won or more (β = .29), and not living with the patient (β = .29), as well as an income level of 3 to less than 5 million won (β = .18), were associated with a higher health-related QOL. Conversely, greater health and psychological problems (β = -.37) and religious/spiritual support (β = -.20) were linked to a lower health-related QOL.

### Interaction effect of age and relationship with patient on health-related QOL

This study examined the interaction effect between the age of caregivers and their relationship with patients on health-related QOL, considering the family-centered sociocultural characteristics of Korea. The analysis employed Model 1 of the Process Macro proposed by Hayes [21], and the results are presented in Table 5. The relationship with the patient was dummy-coded as spouse and non-spouse based on the results of univariate analysis. Age was mean-centered before being simultaneously entered into the regression model. The results revealed a significant interaction term between age and relationship with the patient (B = -0.024, p = .049). The explanatory power (R²) of this research model was.241, indicating that age and relationship with the patient accounted for 24.1% of the variance in health-related QOL. Moreover, the increase in R² (.024) due to the addition of the interaction term was statistically significant (F = 3.95, p = .049).

**Table 5:** Interaction effect of ages and relationship with patient on health-related quality of life.

Variables	B	SE	t (p)	LLCI	ULCI
**Age**	-0.012	0.006	-2.23 (.028)	-0.023	-0.001
**Relationship with patient (spouse)** ^*^	1.142	0.733	1.56 (.122)	-0.309	2.593
**Age × Relationship with patient (spouse)** ^*^	-0.024	0.012	-1.99 (.049)	-0.047	-0.0001
R² = .241, F (p) = 13.22 (<.001)**/** △R² = .024, F (p) = 3.95 (.049)					

*Dummy variables of reference group: Relationship with patient (Non-spouse)

LLCI= Lower Limit Confidence Interval; ULCI= Upper Limit Confidence Interval.

**Fig 2:**
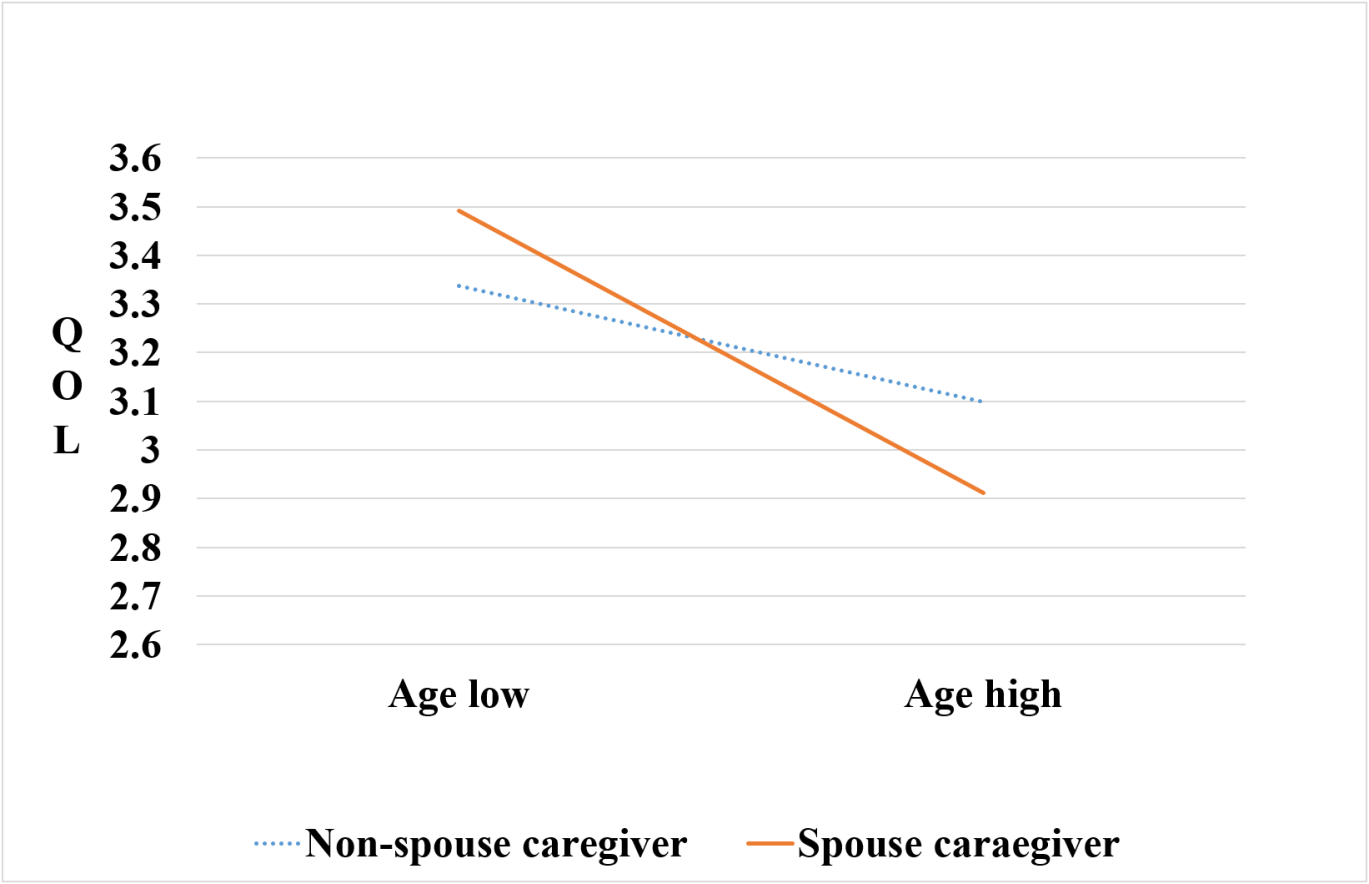
Simple slope analysis of the interaction.

The simple slopes of health-related QOL for spouse and non-spouse caregivers in relation to age are presented in Table 6 and Fig 2. For non-spouse caregivers, there was a slight tendency for health-related QOL to decrease as age increased (B = -0.012, p = .028). For spouse caregivers, the decrease in health-related QOL was more pronounced and statistically significant as age increased (B = -0.036, p = .001).

**Table 6:** Conditional effects of age on health-related quality of life by relationship with patient.

Variables		B	SE	t (p)	LLCI	ULCI
**Relationship** **with patient**	**Non-spouse**	-0.012	0.006	-2.23 (.028)	-0.023	-0.001
	**Spouse**	-0.036	0.011	-3.39 (.001)	-0.057	-0.015

LLCI= Lower Limit Confidence Interval; ULCI= Upper Limit Confidence Interval.

## Discussion

This study was undertaken to understand the unmet needs and health-related QOL of family caregivers who provide the majority of care for cancer patients. By determining how unmet needs influence the health-related QOL of family caregivers, this study aimed to generate essential information for the development of targeted nursing interventions designed to enhance their well-being.

The unmet needs score for the participants in this study was found to be 1.75 points. These results are slightly higher than the 1.57 points reported by Choi [18]and the 64.77 points (equivalent to 1.62 points) reported by Lee and Cha [13]. This discrepancy is likely due to the fact that the participants in the studies by Choi [18] and Lee & Cha [13] were family members of cancer patients hospitalized in hospice facilities. While patients in hospitals or specialized medical institutions receive care from various medical professionals, even in cases of severe illness, the unmet needs in this study were higher for outpatients. This is because family caregivers are required to take on the responsibility of patient care. Indeed, the highest demand among the unmet needs of the study participants was for medical staff support, which corroborates this finding.

In the sub-area of unmet needs, the demand related to healthcare staff emerged as the top priority, aligning with the findings of most studies on family caregivers in Korea [3,13,18]. This priority reflects the reality that family caregivers, often lacking professional knowledge and skills, are responsible for all support and decision-making related to the patient’s treatment [9]. Consequently, there is a recognized need for prompt communication and intervention with healthcare staff in this complex treatment landscape.

Next to the needs related to healthcare staff, the unmet need for information among caregivers was high, aligning with findings from several previous studies [2,3,7,9,13,18], which identified the primary needs of caregivers as information regarding the disease and its treatment. Caregivers reported a high demand for information and education regarding caregiving skills such as symptom management, medication administration, toileting, and handling equipment [22]. A lack of knowledge about the disease can lead to vague anxiety in caregivers, diminishing their problem-solving abilities and adversely affecting patient care [5]. Consequently, it is crucial to continuously provide family caregivers of cancer patients with appropriate information and relevant education. A meta-analysis by Northouse et al. [23] found that interventions addressing information needs were the most effective among 29 interventions designed to resolve unmet needs of caregivers. Standardized educational materials, online information platforms, and 24-hour counseling services have been shown to contribute to reducing caregiver burden. In Korea, the home-based hospice pilot program, which has been providing symptom management and 24-hour telephone counseling services since 2016, has received positive feedback, with a satisfaction rate of 91.3% among bereaved caregivers [24]. Therefore, efforts should be made to expand specialized institutions for home-based hospice care to strengthen caregiver support systems and to operate standardized education and information programs centered on medical institutions and communities to provide more systematic and effective support.

The unmet needs of the study participants varied according to the educational background of their caregivers. This aligns with the findings of Lee et al. [3], who reported that caregivers with less than a middle school education had higher unmet needs than those with high school or college educations. Lower educational levels were associated with higher levels of unmet needs. This may be attributed to the fact that caregivers with lower education levels are likely to have lower incomes or fewer opportunities to access various forms of information and support. Furthermore, the unmet needs of caregivers were found to be greater for those caring for stage 3 cancer patients than for those caring for stage 1 cancer patients. As the cancer stage advances, the severity of the patient’s symptoms increases, naturally leading to greater unmet needs for the caregiver.

In this study, the health-related QOL score for family caregivers of cancer patients was 3.08 points. This score is known to be lower than that of the general population [8], yet it is comparable to the scores of caregivers for other chronic conditions. Specifically, it is similar to the 3.10 points reported for caregivers of cancer patients in hospice specialized institutions [18], the 3.19 points for caregivers of stroke patients living in the community [25], and the 3.05 points for caregivers of patients with severe mental illness [12].

In the current study, regarding the impact of general characteristics on health-related QOL, it was observed that higher age groups, lower income levels, and lower education levels were associated with lower health-related QOL. These findings align with the well-established factors known to influence QOL [4,12,18,26]. However, marital status presented an unexpected trend: married individuals reported a lower QOL compared to those who were unmarried, divorced, or widowed. This is contrary to previous research [3,12], which suggested that having a spouse can improve QOL by providing psychological stability and happiness. Li et al. [27] offered an explanation for this discrepancy, noting that spouses of cancer patients often assume the role of primary caregiver, which can be more distressing than the roles of other caregivers, particularly during the patient’s end-of-life phase. Indeed, in this study, spouses constituted the largest group of caregivers (41.9%) and experienced a significantly lower QOL in their relationship with the patient compared to that of parents and other relatives.

In the present study, unmet needs exhibited a significant negative correlation with health-related QOL, corroborating the findings of prior research, which indicated that higher unmet needs are associated with a lower QOL [3,8,9,18,26]. Furthermore, the study confirmed that the QOL of caregivers is influenced not only by physical, psychological, social, religious, and spiritual needs but also by needs related to facilities and healthcare staff. These findings suggest that the domain of needs impacting the QOL of cancer patient caregivers is multifaceted, necessitating the provision of multidisciplinary management services to address these issues. Given the established importance of multidisciplinary treatment for cancer patients [2], it is imperative to recognize the need for multidisciplinary services when developing policies or nursing intervention strategies for family caregivers.

The factors influencing the health-related QOL of the participants in this study included health and psychological problems, living with patients, income level, and religious/spiritual support. Of these, the unmet needs of health and psychological problems were identified as the most significant variables affecting QOL. Previous studies have also highlighted the importance of care providers’ health and psychological problems as major factors influencing QOL [4,5,26]. Given et al. [4] found that caregivers of cancer patients not only experienced changes in appetite and general physical symptoms such as headaches, fatigue, and insomnia but also often neglected their own chronic conditions, including high blood pressure, diabetes, and heart disease, and increased their use of cigarettes and alcohol. Furthermore, a study by Sklenarova et al. [28] revealed that caregivers experienced higher levels of distress and anxiety than the patients themselves. These psychological burdens were reported to reduce their QOL by increasing the overall burden of care [3,4]. Moreover, because families continuously exchange energy, resources, and emotions within the family system, the physical and mental health of caregivers is interconnected with that of cancer patients [29–31].

In the context of family caregiving for cancer patients, studies have demonstrated a clear bidirectional relationship between caregiver well-being and patient outcomes. Increased caregiver burden can negatively impact the quality of care provided, leading to feelings of anxiety and guilt in patients [11,30]. Conversely, a patient’s declining health can exacerbate caregiver stress and health problems [31]. Recognizing this interconnectedness, the World Health Organization advocates for treating patients and caregivers as a unified entity, suggesting dyadic interventions as a promising approach. Dyadic interventions, which emphasize the mutual influence between patients and caregivers, actively involve both parties in the treatment and care process. Research has shown that such interventions can alleviate patient symptoms, enhance caregivers’ QOL, and reduce caregiver fatigue and depression [30–32]. For instance, Milbury et al. [31] found that a dyadic yoga program not only decreased cancer symptom severity and depression in patients but also improved caregiver mood, fatigue levels, and mental well-being. Similarly, Elis et al. [33] found that caregiver involvement in health-promoting behaviors with patients, such as exercise and diet, significantly improved adherence to these behaviors and enhanced patient safety during exercise. A range of other dyadic interventions, including psychosocial support, mindfulness training, coping skills, and creative arts therapies, have also demonstrated effectiveness in improving QOL for both patients and caregivers [32]. Given the strong tradition of family-centered care in Korea, integrated support programs that incorporate dyadic interventions are likely to be particularly effective. Future research should focus on developing and evaluating culturally tailored dyadic interventions to optimize outcomes for both patients and caregivers in Korea.

Next, the health-related QOL was observed to be lower among caregivers living with patients, which is likely attributable to the heightened physical burden and stress associated with managing various and demanding caregiving roles. Given et al. [4] identified long-term caregiving—defined as providing care for over 1,000 hours per year—as a factor that can negatively impact caregivers’ QOL. However, while Korean society is losing its influence, it still remains a family-centered society where many patients live with their families. In this study, more than half (62.0%) of the caregivers were living with the patients. Many social systems in Korea are also designed based on family structures rather than individuals, which imposes a strong emphasis on the survival and functioning of family units [34]. Therefore, the occurrence of cancer patients within families in Korean society not only deteriorates the QOL of caregivers but also leads to relational conflicts surrounding caregiving among family members. Such relational conflicts can result in family disintegration and instability [29,34].

In a qualitative study [29], it was reported that when family members caring for cancer patients are unable to fulfill their developmental tasks, they experience feelings of burden and victimization, which can escalate into conflicts within the family when they do not receive help with caregiving from other family members. Moreover, recent Korean society has seen a rapid increase in single-person households and simplification of generations, leading to a rise in small and nuclear families. This change in the family social environment raises the possibility of caregiving gaps due to the weakening of family caregiving environments [35]. As a response to the low birthrate and aging society over the past two decades, social care has been expanded, with national policies such as “free childcare” and “long-term care insurance for the elderly” introduced to reduce the burden of caring for children and elderly individuals with dementia. Various other policies have also been proposed and are showing effects [35]. Current national policies for cancer patients are largely confined to medical insurance and counseling/psychological support, with a notable absence of comprehensive policies and awareness concerning family caregivers [36]. It is therefore essential to reinforce governmental commitment by incorporating cancer patients into national social care initiatives. This should include the provision of services comparable to respite care, hospice assistance, and homemaker support, to ensure the continuity of care in the home setting and improve the QOL for those providing that care.

Income level was another factor that influenced the health-related QOL for caregivers of cancer patients, with higher income correlating to a higher health-related QOL. Cancer imposes a socio-economic burden due to the limitations it places on the socioeconomic activities of both patients and their families, as well as the costs associated with treatment and disease management. Consequently, lower income levels can exacerbate the financial burden on patients and their families, leading to a diminished QOL [9,18]. Therefore, recognizing that current national policies for cancer patients, such as the Special Calculation Exemption System, are primarily focused on medical expenses [37], there is an urgent need for comprehensive financial support policies to alleviate the substantial economic burden on families. A key step is to explore the possibility of including essential caregiving costs within cancer treatment expenses, thereby enabling public health insurance to cover a portion of these expenses. Furthermore, accessibility to social welfare services and sponsoring organizations should be enhanced, and related laws should be amended to create a more systematic financial support framework that provides tailored assistance based on income and living environment.

Next, higher unmet needs of religious/spiritual support were associated with lower health-related QOL for caregivers. Cancer patients and caregivers both experience a vague sense of anxiety and fear of death [29]. However, religious support has been shown to help individuals manage anxiety and depression, fostering a more positive outlook on life [4,38]. According to Given et al. [4], caregivers who received spiritual support experienced reduced stress levels and an enhanced sense of well-being. Similarly, a study by Kim & Choi [38] found that the QOL was higher for families of terminal cancer patients when their spiritual needs were addressed. Consequently, there is a clear need to evaluate and address the religious and spiritual needs of caregivers of cancer patients by connecting them with spiritual leaders or religious counseling. Additionally, it is essential to develop and offer a robust intervention program aimed at improving their spiritual well-being.

Lastly, considering the socio-cultural characteristics of Korea’s family-centered society, we explored the interaction between age and the caregiver’s relationship to the patient. The results showed that for spousal caregivers, compared to non-spousal caregivers, the negative impact on health-related QOL associated with increasing age was greater. Older caregivers face not only their own health issues but also the added responsibilities of caregiving. In particular, in spousal relationships, emotional bonds are accompanied by psychological burdens that become more pronounced with increasing age [39]. These findings suggest that the health-related QOL is not solely determined by the caregiver’s relationship with the patient; rather, it reflects a complex phenomenon where caregiving methods and demands evolve with age.

Additionally, in Korean society—where nuclear family structures and shifting values regarding caregiving make it increasingly difficult for children to care for their parents [34]—this highlights the importance of policy alternatives to address these challenges. However, most research on caregivers has focused on those caring for elderly individuals with dementia [34,39–41], resulting in a relative lack of studies on caregivers for cancer patients. Therefore, future research should more comprehensively examine the various factors affecting the QOL of cancer patient caregivers. This will enable a deeper understanding of how physical, emotional, and social factors interact and help develop effective support strategies.

## Conclusion

This study aimed to identify the unmet needs and health-related QOL of family caregivers who primarily support cancer patients, and to analyze the influence of unmet needs on health-related QOL. The findings revealed that the greatest unmet need for these caregivers was in the area of information and education, and a significant negative correlation was found between unmet needs and overall QOL. Key factors influencing caregiver QOL included health management and psychological needs, cohabitation with the patient, income level, and religious/spiritual needs. Notably, QOL tended to decrease with increasing caregiver age and when the caregiver was a spouse. Based on these results, several practical measures are recommended to address the unmet needs of cancer patient caregivers. First, to meet the need for information and education, systematic programs should be implemented within healthcare institutions and communities to provide comprehensive information on patient symptom management, emergency response strategies, and essential nursing skills; accessibility to both online and offline educational platforms should also be expanded. Second, to address health management and psychological needs, support systems such as respite care and hospice aide services are crucial to alleviate caregiver burden; furthermore, access to professional psychological counseling and the facilitation of self-help groups are needed to mitigate mental stress and depression—the development and evaluation of physical and psychological intervention programs utilizing dyadic approaches are particularly encouraged. Third, to address economic needs, comprehensive financial support policies are essential to reduce the financial strain on cancer patient families, and consideration should be given to incorporating essential care costs into standard cancer treatment coverage. Fourth, to address spiritual needs, collaboration with community religious organizations is recommended to provide visiting counseling services from religious leaders, as well as grief and bereavement support services for families of terminally ill cancer patients.

Nonetheless, this study has limitations. First, this study utilized convenience sampling, which may introduce potential selection bias. As a result, the generalizability of these findings should be interpreted with caution. Moreover, the study focused solely on family caregivers of patients receiving outpatient services at a general hospital in one region, which cautions against generalizing these findings to all cancer patient caregivers. Additionally, while our sample size of 129 participants was calculated to be sufficient for statistical analysis, we recognize it may not fully capture the diverse range of experiences among family caregivers. Therefore, future research should consider employing probability sampling methods to enhance the representativeness of the sample and minimize potential bias, and recruit a sufficient number of participants from various regions to better reflect the diversity of caregivers. This approach will help improve the generalizability of the findings and provide a more comprehensive understanding of the factors affecting family caregivers. Second, unmet needs and health-related QOL were measured using self-report methods in this study, which may have been influenced by respondents’ personal subjectivity or social expectations. Future studies could consider incorporating multiple data collection methods, such as combining self-reports with objective measures or qualitative interviews, to provide a more comprehensive and potentially more accurate assessment of caregivers’ experiences. Third, its cross-sectional design limited the ability to fully understand the characteristics and causal relationships of the multidimensional aspects of the caregiving experience. Therefore, we have highlighted the need for longitudinal studies to provide valuable insights into how the unmet needs and health-related QOL of family caregivers for cancer patients evolve over time. Finally, the reliability of the religious/spiritual needs subscale in the unmet needs instrument (CANT-C) was relatively low at.60. This is likely because 50.4% of the participants reported no religious affiliation, potentially leading to ambiguous and inconsistent responses to questions about religious/spiritual needs. Future research should consider developing more specific questionnaire items that take into account diverse religious backgrounds to reassess religious/spiritual needs.

Despite these limitations, this study was conducted at a large general hospital visited by patients from various regions, and the use of validated survey instruments helped mitigate these limitations. Furthermore, the study has significance in specifically identifying the unmet needs of cancer patient family caregivers and providing implications for effective support programs and policies to improve their QOL.

## Supporting information

S1 Dataset Raw data for experimental results and statistical analysis. This file contains the minimal dataset used to reach the conclusions drawn in the manuscript with related metadata and methods, and any additional data required to replicate the reported study findings. Data is provided in CSV format.
(SAV)
